# Effect of Arsenic Exposure on NRF2-KEAP1 Pathway and Epigenetic Modification

**DOI:** 10.1007/s12011-017-1219-4

**Published:** 2017-12-15

**Authors:** Beata Janasik, Edyta Reszka, Magdalena Stanislawska, Ewa Jablonska, Renata Kuras, Edyta Wieczorek, Beata Malachowska, Wojciech Fendler, Wojciech Wasowicz

**Affiliations:** 1 Department of Environmental and Biological Monitoring, Nofer Institute of Occupational Medicine, St. Teresy 8, 91-348 Lodz, Poland; 2 Department of Genetics and Epigenetics, Nofer Institute of Occupational Medicine, Lodz, Poland; 3 Department of Biostatistics and Translational Medicine, Medical University of Lodz, Lodz, Poland; 4 Studies in Molecular Medicine, Medical University of Warsaw, Warsaw, Poland

**Keywords:** Arsenic, NRF2, KEAP1, Cytoprotective genes, Gene expression, Methylation

## Abstract

Arsenic (As) is a known toxic element and carcinogen. Transcription factor nuclear factor-erythroid 2-related factor 2 (NRF2) controls cellular adaptation to oxidants and electrophiles by inducing antioxidant genes in response to redox stress. To explore associations between As level and NRF2-regulated cytoprotective genes expression, an observational study was conducted in a population of 61 occupationally exposed men with median (Me) age 50 years (interquartile range (IQR) 42–54) and in a control group of 52 men aged 40 (IQR 31–51.5) without occupational exposure. *NRF2*, *KEAP1*, *GSTP1*, *HMOX1*, *NQO1*, *PRDX1*, and *TXNRD1* transcript levels were determined by means of quantitative real-time PCR along with the gene expression, methylation of *NRF2* and *KEAP1*, as well as global DNA methylation were assessed. The median urine As _tot._ level in the exposed and control group was found to be 21.8 μg/g creat. (IQR 15.5–39.8 μg/g creat.) and 3.8 μg/g creat. (IQR 2.5–9.3) (*p* < 0.001). Global DNA methylation was significantly higher in occupationally exposed workers than in controls (Me 14.1 (IQR 9.5–18.1) vs Me 8.5 (IQR 5.9–12.6) *p* < 0.0001). *NRF2* mRNA level was positively correlated with expression of all investigated NRF2-target genes in both groups (0.37 > *R* < 0.76, all *p* values < 0.0001). The multivariate linear regression adjusting for global methylation showed that As(III) level was significantly associated with expression of *TXNRD1*, *GSTP1*, *HMOX1*, and *PRDX1*. The results of this study indicate that arsenic occupational exposure is positively associated with global DNA methylation. The findings provide evidence for rather inactivation of NRF2-KEAP1 pathway in response to chronic arsenic exposure.

## Introduction

The factors present in the human environment significantly affect the integrity of the genetic material. Many of them have mutagenic and even carcinogenic effects. An important group of compounds with carcinogenic effect, which occur both in the living and occupational environment, are metals such as arsenic. This metaloid is classified by the International Agency for Research on Cancer [1], the Agency for Toxic Substances and Disease Registry [2], as a human carcinogen. The classification is based on epidemiological studies confirming the increase in the incidence of the lung cancer risk for inhalation exposure and skin and bladder cancers in people exposed to arsenic ingestion [3–5]. The main theory underlying the mechanisms of carcinogenesis of this factor is the role of oxidative stress [6–8]. Oxidative stress is the imbalance between prooxidant and antioxidant compounds/mechanisms in a cell resulting in an increase of prooxidative events [9]. This initiates a pathogenetic process which may ultimately lead to carcinogenesis. Abnormalities in the cells caused by oxidative stress are closely associated with the increased risk of cancer as a result of DNA damage, genomic DNA hypomethylation and hypermethylation, and changes in the regulatory mechanisms of cell proliferation and apoptosis. [6, 7, 10–12]. The antioxidant response is one of the most effective cellular defense mechanisms. Recent studies indicate that the main role in balancing the redox cell disorders and related consequences of exposure to inorganic arsenic (iAs) is played by transcription factor *NRF2* (nuclear erythroid 2-related factor) linked to the antioxidant response element (ARE) of the nuclear DNA through a leucine zipper motif (bZip) [13]. The conducted genetic and biochemical studies confirm the importance of *NRF2* in the transcriptional regulation of the ARE-dependent genes. *NRF2* activates proteins with cytoprotective action deactivating electrophilic metabolites and reactive oxygen species stabilizing the redox potential. These include inter alia, glutathione S-transferase (GST), NAD(P)H-quinone 1 (NQO1) reductase, epoxide hydrolase, glutathione reductase, catalase, and superoxide dismutase [6, 7, 10, 11]. The main path that is responsible for the response to oxidative stress and the regulation of the activity of the *NRF2* factor is that of *KEAP1-NRF2-ARE*. KEAP1 (Kelch-like ECH-associated protein) is responsible for cytoplasm-nuclear transport and proteasome destruction of *NRF2*. Under the conditions of homeostasis, the transcription factor *NRF2* remains in the cytoplasm through forming a complex with KEAP1. When redox disorders occur, in response to the activity of the redox balance disrupters, *NRF2* protein is released from the *NRF2-KEAP1* complex and moves from the cytoplasm to the nucleus by activating the proper genes encoding proteins with cytoprotective action. The final result is an increased duration of *NRF2* and increased expression of the ARE-dependent genes. Therefore, *NRF2* is a protein referred to as necessary in the process aimed at protecting the cells against genetic damage under the effects of carcinogens [6, 7, 10, 11]. However, recent research attempts showed the “dark side” of the transcription factor *NRF2*. Independent studies indicated the responsibility of *NRF2* for the processes of increased survival of tumor cells and stimulation of the carcinogenesis process [14, 15]. Mutations and epigenetic modifications affect the regulation and fate of *NRF2*, inter alia by disrupting the interactions between *NRF2-KEAP1* and affecting *NRF2* protein stabilization—these disturbances may ultimately enhance/reveal tumor phenotypes [14, 15]. Furthermore, transcription factor *NRF2* over-expression caused by intense induction by xenobiotics can be a carcinogenic factor. Ikeda et al. [16], for the first time, described the negative impact of *NRF2*, pointing to its role in the development of liver cancer in animal experiments. Also, a significant impact is indicated on the part of the *NRF2*-dependent genes on the process of carcinogenesis. Heme oxygenese 1 enzyme (HO1 coded by *HMOX1* gene), similar to *NRF2* in normal homeostasis, exhibits antioxidant effects, while over-expression has been observed in different types of cancer changes [17]. In both cases, *NRF2*-dependent antioxidant response is indicated as a factor supporting the neoplastic processes by excessive induction mechanism *NRF2-KEAP1* and epigenetic modifications. It seems interesting to assess the impact of exposure to inorganic arsenic in the conditions of occupational exposure on the expression of the transcription factor *NRF2*, *NRF2*-dependent genes, and ARE-dependent genes. The evaluation of these factors in a specific exposure related to specific lifestyle factors and professions will make it possible to better understand the potentially increased risk of tumors associated with the exposure to these metals. Therefore, the aim of the study was to investigate the association between iAs level in a population with occupational exposure to As and the link between iAs levels and *NRF2*-dependent genes (*GSTP1*, *HMOX1*, *NQO1*, *PRDX1*, *TXNRD1*) and others associated with redox homeostasis and epigenetic modification.

## Materials and Methods

### Study Population

This study was performed on copper mill workers (males *n* = 61) from the southwestern part of Poland with a median age of 50 years (interquartile range (IQR) 42–54 years). The median body mass index (BMI) was 28.1 kg/m^2^ (IQR 25.0–30.7 kg/m^2^). Non-occupationally exposed healthy controls (*n* = 52)—males with median age 40 years (IQR 32–51.5 years)—were recruited. The median body mass index in controls equaled 26.6 kg/m^2^ (IQR 24.4–28.4 kg/m^2^) (Table 1). The Institutional Ethics Committee for Scientific Research approved the study protocol and a written consent was obtained from each participant of the study.

**Table 1:** Characteristics of examined subjects

	Exposed group	Control group	*p* values
	Median (IQR)	Median (IQR)	
Age [years]	50.00 (42.00–54.00)	40.00 (32.00–51.50)	0.0032
BMI [kg/m^2^]	28.09 (25.01–30.72)	26.59 (24.42–28.35)	0.0286
Job seniority [years]	27.00 (18.00–35.00)	NA	NA
	N/N total (%)	N/N total (%)	
Smoking	19/61 (31.15%)	15/52 (28.85%)	0.7904
Fish	14/57 (24.56%)	15/52 (28.85%)	0.7729

*NA* not applicable

### Arsenic Determination

Arsenic in the air and arsenic in urine were determined using inductively coupled plasma mass spectrometry (ICP-MS) as previously described by Janasik et al. [18]. Air samples were collected via the individual dosimetry method in the breathing zone of each worker continuously throughout a 6–7-h period of time. To determine arsenic and its compounds in the air of the workplace, ICP-MS was applied (version offered by NIOSH Manual of Analytical Methods, Fourth Edition Method 7301, Issue 1, 2003, Elements by ICP (Aqua Regia Ashing) and Method 7901, Issue 2, 1994, Arsenic Trioxide, as As) [19].

### Analyses of As in Urine (As-U)

Urine samples were collected from the exposed group as well as from the controls. Workers provided spot urine samples immediately after the shift-end on the second day after exposure. The control subjects provided spot urine samples collected on the morning of arrival to the laboratory. Prior to dilution, the samples were centrifuged at 4000 rpm for 10 min and then the supernatant was diluted tenfold with 1.0% HNO_3_ for the total arsenic and the mobile phase for the speciation analyses. ELAN DRC-e ICP-MS with a dynamic reaction cell (Perkin Elmer, SCIEX, USA) was used for arsenic determination. The instrument Series 200 HPLC (Perkin Elmer, SCIEX, USA) was applied to separate arsenic chemical forms. The precision of the method for determination the individual forms of arsenic was as follows: AsB, 3.4%; As(III), 4.2%; DMA, 1.9%; MMA, 1.8%; and As(V), 2.3%. Accuracy measurement as the difference between the measurement and the accepted value for NIST 2669 was as follows: AsB, 1.1%; As(III), 1.5%; DMA, 2.2%; MMA, 3.9%; and As(V), 3.5%.

Certified reference material SRM 2669 (human urine) from the National Institute of Standard and Technology (NIST) was examined at the beginning and at the end of the analysis. The laboratory participates in the external quality program for the total arsenic determination organized by the Institute of Occupational Social and Environmental Medicine of the University of Erlangen, Nuremberg (G-EQUAS).

### Gene Expression Analysis

*NRF2*, *KEAP1*, and five *NRF2*-targets selection: *GSTP1*, *HMOX1*, *NQO1*, *PRDX1*, and *TXNRD1* were made according to studies data where associations between arsenic and *NRF2*-regulated genes were observed [20]. Total RNA was isolated from venous blood using PAXgene RNA Blood Mini Kit (PreAnalytiX GmbH, Hombrechtikon, Switzerland). Transcript levels in peripheral blood leukocytes were determined by means of quantitative real-time PCR (qPCR) with *GAPDH* and *RPLP0* reference genes. Primer sequences for target genes were presented in a previous study [21]. The cDNA was synthesized with Transcriptor First Strand cDNA Synthesis Kit (Roche, Basel, Switzerland). All the samples were amplified in duplicate. Expression was quantified with FastStart Essential SYBR Green Master (Roche, Basel, Switzerland) using the LightCycler® 96 System (Roche, Basel, Switzerland). Gene expression data were evaluated by dCt method with reference genes-normalized relative quantification.

### NRF2, KEAP1 Promoter Methylation

Promoter regions with transcriptional start site and first exon of NRF2 and KEAP1 were analyzed with transcriptional start sites DNA sequence: DBTSS database (http://dbtss.hgc.jp) for further CpG islands identification. Chemical modification of isolated genomic DNA was performed with Cells-to-CpG™ Bisulfite Conversion Kit (Thermo Fisher Scientific). DNA methylation of NRF2 and KEAP1 was analyzed using quantitative methylation-specific real-time PCR assay (qMSP) with FastStart Essential SYBR Green Master (Roche, Basel, Switzerland). Two sets of qMSP primers were designed using Methyl Primer Express ® Software v1.0 (Thermo Fisher Scientific, Carlsbad, CA, USA). The methylated (M) primers are 5′ TAACGTTTTTTCGGGGTTTC 3′(NRF2 F), 5′ CTCCGTTAACTCCCCGATAC 3′ (NRF2 R), 5′ TTTTTTTTAGATTTTGCGGTC 3′(KEAP1 F), and 5′ ATCTCCCGATTTCGTTACTAA 3′(KEAP1 R). The unmethylated (U) primers are 5′ TATTAATGTTTTTTTGGGGTTTT 3′(NRF2 F), 5′ CTCCATTAACTCCCCAATACCAA 3′(NRF2 R), 5′ TAATTTTTTTTAGATTTTGTGGTT 3′ (KEAP1 F), and 5′ CCATCTCCCAATTTCATTACTAAA 3′(KEAP1 R). A methylation index (MI) expressed as percentage of gene methylation was calculated for each sample using the following formula: MI = [1 / (1 + 2^−(CtU−CtM)^] × 100% [22].

### Global DNA Methylation

Global DNA methylation levels were assessed by colorimetric ELISA method using Methylflash Methylated DNA Quantification Kit (Epigentek, Farmingdale, NY, USA), according to manufacturer’s instructions. One hundred nanograms of DNA isolated from blood was used for analyses. Each sample was analyzed in duplicates. The calculation of 5-methylcytosine amount was done with the use of standard curve created using defined dilutions of methylated genomic DNA. Methylation levels were calculated relative to the methylated control DNA (included in the kit) and expressed as a percentage of total methylated DNA. The intrassay CV was 8.8%.

### Statistical Analysis

For measurable parameters, the authors calculated median values with interquartile ranges. For non-measurable values, a number of observations with respected percentage were given. The Chi-square test for independent pairs was used with Yates correction or Fisher’s exact test, which were used if necessary. Mann-Whitney’s *U* test was used for non-paired categorical and continuous data analysis. Correlation coefficients were calculated with Spearman rank test. Linear regression models were performed in order to evaluate the relationship between gene expression values and As(III) level standardized to creatinine level with adjustment to methylation level. *p* values lower than 0.05 were considered statistically significant. Statistical analyses were done with STATISTICA 12.5 PL (StatSoft, Tulsa, OK, USA).

## Results

The airborne arsenic concentrations (As-A) in occupational settings, measured using individual sampling, varied from 2.8 to 34.4 μg/m^3^ (mean 9.5 μg/m^3^). The occupational and environmentally (control) exposed group differed significantly in concentrations of arsenic in urine expressed both in microgram per liter and microgram per gram creatinine (Table 2). The mean creatinine values in urine of the exposed and control group were respectively 1.3 ± 0.64 g/l and 1.2 ± 0.63 g/l. According to the World Health Organization and American Conference on Governmental and Industrial Hygienist (ACGIH), urine specimens that were highly dilute or highly concentrated (creatinine concentration: > 0.3 g/L and < 3.0 g/L) were discarded and another sample was collected [23]. There was no significant correlation between the age and BMI and between methylation and gene expression regardless of the group.

**Table 2:** Comparison of arsenic urinary concentration between groups

	Exposed group	Control group	*p*
	Median (IQR)	Median (IQR)	
As-total (μg/l)	29.50 (14.70–54.70)	4.60 (2.55–15.90)	< 0.0001
As-total (μg/g creat.)	21.83 (15.49–39.77)	3.75 (2.52–9.26)	< 0.0001
As(III) (μg/l)	3.10 (1.90–6.40)	0.50 (0.19–0.70)	< 0.0001
As(III) (μg/g creat.)	2.30 (1.50–4.90)	0.36 (0.16–0.59)	< 0.0001
As-A (μg/m^3^)	9.45 (2.80–34.35)	NA	NA
iAs (μg/l)	4.50 (2.20–9.20)	0.50 (0.17–0.95)	< 0.0001
iAs (μg/g creat.)	3.50 (2.10–6.90)	0.37 (0.13–0.69)	< 0.0001

*NA* not applicable

### Gene Expression

**Fig. 1:**
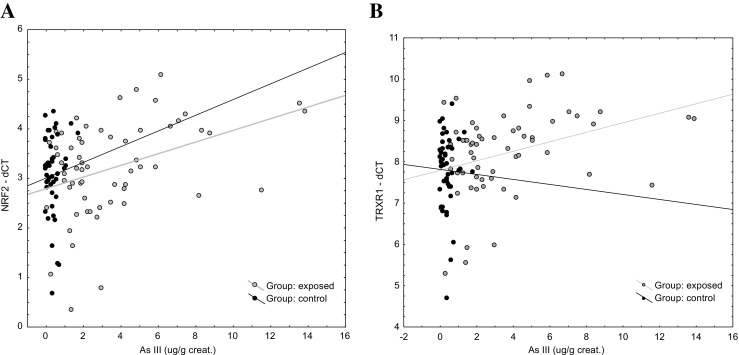
Example of relationship between As(III) concentration in urine and NRF2 (**a**) and TRXR1 (**b**) expression

No statistically significant differences in NRF2-KEAP1-ARE pathway associated genes between exposed workers and controls were found. However, in the occupationally exposed workers group, the statistically significant relationship between NNR2 target genes and As(III) concentrations was found. A similar relationship was found between expression of NRF2 target genes and the sum of inorganic arsenic concentration (Table 3). *NRF2* mRNA level was positively correlated with expression of *NRF2* targets (Table 3) and with KEAP 1 (*R* = 0.72, *p* < 0.0001). KEAP1 mRNA level was positively associated with investigated gene expression (Table 4). Multivariate linear regression adjusting for global methylation indicated significant relationships between As(III)-U level standardized to creatinine and expression of *TRXR1*, *GSTP1*, *HMOX1*, and *PRDX1* at the margin of statistical significance (Table 5, Fig. 1).

**Table 3:** Association between NRF2-regulated gene expression and arsenic concentrations in urine

Exposed	NRF2	KEAP1	HMOX1	GSTP1	NQO1	PRDX1	TRXR1
As tot. (μg/l)	0.17	0.08	0.13	0.06	0.01	0.11	0.18
As(III) (μg/l)	0.29	0.22	0.31	0.24	0.15	0.28	0.35
As(V) (μg/l)	0.16	0.15	0.16	0.14	0.06	0.27	0.18
DMA (μg/l)	0.11	0.04	0.07	0.07	− 0.01	0.13	0.12
MMA (μg/l)	0.24	− 0.01	0.01	− 0.01	− 0.01	0.13	0.19
AsB (μg/l)	0.19	0.10	0.16	0.12	0.02	0.07	0.18
As-A (μg/m3)	− 0.03	− 0.14	− 0.01	0.00	0.07	0.06	0.02
Sum iAs+MMA (μg/l)	0.28	0.13	0.22	0.15	0.09	0.25	0.31
Sum iAs (μg/l)	0.27	0.21	0.29	0.22	0.13	0.30	0.32
Sum iAs+MMA+DMA (μg/l)	0.19	0.10	0.14	0.11	0.04	0.18	0.20
Control	NRF2	KEAP1	HMOX1	GSTP1	NQO1	PRDX1	TRXR1
As tot. (μg/l)	− 0.12	− 0.19	− 0.09	− 0.14	− 0.16	− 0.16	− 0.20
As(III) (μg/l)	− 0.08	− 0.15	− 0.18	− 0.12	− 0.21	− 0.17	− 0.18
As(V) (μg/l)	0.31	0.17	0.11	0.38	− 0.18	0.34	0.09
DMA (μg/l)	− 0.10	− 0.19	− 0.08	− 0.11	− 0.18	− 0.17	− 0.17
MMA (μg/l)	− 0.12	− 0.22	− 0.25	− 0.23	− 0.15	− 0.24	− 0.18
AsB (μg/l)	− 0.13	− 0.17	− 0.06	− 0.04	− 0.10	− 0.13	− 0.19
Sum iAs+MMA (μg/l)	− 0.12	− 0.20	− 0.20	− 0.20	− 0.20	− 0.13	− 0.23
Sum iAs (μg/l)	− 0.10	− 0.19	− 0.19	− 0.18	− 0.22	− 0.12	− 0.23
Sum iAs+MMA+DMA (μg/l)	− 0.11	− 0.18	− 0.09	− 0.14	− 0.16	− 0.17	− 0.19

**Table 4:** Association between NRF2 and NRF2-regulated gene expression

	*NRF2*-dCT		*KEAP1*-dCT	
	*R*	*p*	*R*	*p*
*HMOX1*-dCT	0.66	< 0.0001	0.75	< 0.0001
*GSTP1*-dCT	0.51	< 0.0001	0.69	< 0.0001
*NQO1*-dCT	0.30	0.0013	0.45	< 0.0001
*PRDX1*-dCT	0.48	< 0.0001	0.62	< 0.0001
*TRXR1*-dCT	0.76	< 0.0001	0.69	< 0.0001

*R*, correlation coefficient

**Table 5:** Association between gene expression, methylation, and urine arsenic level in occupational exposure group

	As(III) (μg/g creat.)		5-mC %		NRF2, KEAP1 promoter methylation		As(III) (μg/g creat.) adj. global methylation		As(III) (μg/g creat.) adj. NRF2, KEAP1 promoter methylation	
	*R*	*p*	*R*	*p*	*R*	*p*	*B*	*p*	*B*	*p*
NRF2-dCT	0.35	0.0061	0.20	0.1218	− 0.21	0.1004	0.11	0.0067	0.08	0.0324
KEAP1-dCT	0.32	0.0133	0.25	0.0494	− 0.03	0.8469	0.09	0.0217	0.10	0.0090
HMOX1-dCT	0.36	0.0051	0.28	0.0324			0.08	0.0211		
GSTP1-dCT	0.27	0.0348	0.06	0.6256			0.07	0.0288		
NQO1-dCT	0.22	0.0949	0.26	0.0449			0.06	0.2194		
PRDX1-dCT	0.30	0.0184	0.06	0.6689			0.08	0.0271		
TRXR1-dCT	0.39	0.0019	0.18	0.1728			0.10	0.0159		

*R*, correlation coefficient; *B*, interpretation as *R*

### DNA Methylation

The occupationally exposed group showed a significantly higher degree of *NRF2* methylation (Me 0.47% (IQR 0.29–0.59%) vs Me 0.27% (IQR 0.13–0.47%), *p* = 0.0017), *KEAP1* methylation (Me 0.13% (IQR 0.03–0.20%) vs Me 0.04% (IQR 0.001–0.18%), *p* = 0.0238), and overall 5-mC content (Me 1.41% (IQR 0.95–1.81%) vs Me 0.85% (IQR 0.59–1.26%) *p* < 0.0001) than the control group. Moreover, smokers in the occupational exposure group observed a higher degree of methylation of the 5-mC (Me 1.63% (IQR 1.41–1.99%) vs Me 1.35% (IQR 0.76–1.55%) *p* = 0.0039).

## Discussion

### As-Mediated Activation of NRF2-KEAP1 Antioxidant Pathway

Oxidative stress is the most widely accepted and studied mechanism of arsenic toxicity. Many researchers nowadays have speculated that the activation of the *NRF2-KEAP1-ARE* pathway by arsenic is beneficial and is likely to be an attempt of cells to counteract the damage effects of the metalloid. Arsenic was reported to induce the *NRF2*-dependent antioxidant response, although the detailed mechanism of *NRF2* induction by arsenic has to be further explored [12–15]. Arsenic elicited both a beneficial *NRF2*-dependent antioxidant response and a cell damaging effect. The net outcome in response to arsenic may be dictated by arsenic species, dose, and duration of arsenic exposure. Nevertheless, activation of the *NRF2-KEAP1-ARE* pathway represented the initial attempt to counteract detrimental effects induced by arsenic and to maintain cellular homeostasis, which is the first barrier against the effects of exposure to arsenic. In experimental studies, increased *NRF2* activity was observed in osteoblasts under the impact of arsenic through the transcriptional activation of the genes encoding HO-1, Prx I. Inorganic arsenic(III) strengthens the cellular expression of *NRF2* on the transcription level and activates the expression of the *NRF2*-dependent genes in cell lines of human keratinocytes (HaCaT) [20, 24]. Exposure to inorganic arsenic is a stimulus that causes increased accumulation of nuclear *NRF2*. Zhao et al. [25] found that interactions between *NRF2*, KEAP1, and *NRF1* coordinated the regulation of cellular antioxidant reactions in response to acute exposure to arsenic. The role of *NRF2* in prevention of damage resulting from oxidative stress and prevention of pathogenetic processes is due to the effect of arsenic on the interactions between KEAP1 and E3 subunit of ubiquitine ligase (Cul 3) for *NRF2*. By decreasing Cul3 activity, *NRF2* ultimately inhibits the degradation of the transcription factor in the cytoplasm and increases the lifetime of *NRF2* from about 20 to 200 min [20]. Dinkova-Kostova et al. [26] as well as Eggler et al. [27] revealed that a number of inducers interacted with cysteine thiols of *KEAP1* to inhibit *NRF2* turnover and activate *NRF2*. He and Ma [13] showed that *NRF2* contains evolutionarily conserved cysteine residues critical for response to antioxidant and electrophile inducers including arsenic. Furthermore, *NRF2* cysteines are required for suppression of *KEAP1-*dependent ubiquitination and transcription activation of *NRF2*. Therefore, a dual sensor mechanism, in which both *KEAP1* and *NRF2* recognize inducers, has evolved to ensure a wide range of ligand recognition by *NRF2*. We found that *NRF2* mRNA level was positively correlated with expression of investigated genes and with *KEAP1* mRNA level which may confirm the dual mechanism of inducer recognition, especially because we obtained also positive correlations between *KEAP1* mRNA level and studies genes expression.

Activation of the antioxidant response element in cytoprotective genes is the first barrier that is responsible for detoxification of arsenic. These genes include redox-balancing proteins (heme oxygenase, tioredoxin reductase), both I and II phase detoxification enzymes (*NQO1*, *GST*) and another stress response protein. The conducted studies demonstrate that occupational arsenic exposure rather inactivates *NRF2* and expression of cytoprotective genes, *TXNRD1 GSTP1*, *HMOX1*, *PRDX1* and expression is of interest and was statistically associated with inorganic arsenic urine level (Table 4, Fig. 1). Although *NRF2* pathway activation by iAs has been reported in various cell types [28–30], however, the experimental data in vivo are very limited and not fully elucidated in humans. Several in vitro experiments have indicated that the TRX system is involved in As-induced cell responses. Hansen et al. and Myers et al. [31, 32] reported that the mRNA and protein expression of TXNRD1 are induced in As(III)-treated cells. Rea et al. [33] confirmed that the mRNA level of the selenoenzyme thioredoxin reductase was increased up to 13-fold under analysis of primary keratinocytes treated with arsenite; however, enzyme activities or protein levels of TRXR1 were not analyzed. Another study, monitoring global gene expression in keratinocytes treated with arsenite, revealed increases in mRNA encoding thioredoxin and TRXR1 [33]. These studies confirm that increased ROS generated in long-term exposure to arsenic is the primary mechanism behind the increased rates of cancer in populations with elevated arsenic exposure. Dodmane et al. [34] reported increased gene expression (HMOX1, GPX2, TXNRD1) in 1T1 urotherial cells, HEK001 keratinocytes, and HBE (human bronchial epithelial) cells. However, similar to Bailey et al. [35, 36] emphasize the differences in the results of the analysis of gene expression in vitro and in vivo. Clewell et al. [37] explain these differences, among others suppression of adaptive responses to oxidative stress with chronic exposure. The results of studies of gene expression in the current project with an average working time of exposure to inorganic arsenic 27 years (range 18–40), can justify the thesis of inhibition/suppression of adaptive responses as a result of chronic exposure. To our knowledge, this is the first work on the study of gene expression in the case of occupational exposure to inorganic arsenic. The observed dependence due to the relatively small and specific group should be interpreted with caution and necessarily confirmed by other studies in occupational exposure conditions.

### As and Global DNA Methylation

DNA methylation is one of the most commonly studied epigenetic regulation mechanisms and is involved in the regulation of many biological processes through the regulation of gene expression. Global genomic DNA methylation is a hallmark of many types of cancers. The underlying mechanisms and physiologic consequences of As-induced alterations in genomic DNA methylation are unknown, probably its mechanism of SAM (S-adenosyl methionine) insufficiency and DNMT (DNA methyltransferase) gene expression [38]. Global DNA hypomethylation was found to be a causative factor for arsenic-induced carcinogenicity, which causes aberrant gene expression, in steroid-, apoptosis-, and cell cycle-related genes. The majority of animal and in vitro studies report that arsenic induces global DNA hypomethylation. Animal studies have suggested that As induces hepatic genomic hypomethylation of DNA [25, 39]. Genomic DNA hypomethylation commonly occurs in tumors and transformed cells and is thought to constitute an early event in some cancers [40]. Benbrahim-Tallaa et al. [41] exposed for 29 weeks human prostatic epithelial cells to As(III) in 5 μM concentration and observed genomic DNA hypomethylation, which was accompanied by reduced DNA methyltransferase activity.

However, instances have been reported of arsenic inducing global hypermethylation. Mass and Wang [42] and Davis et al. [43] observed global DNA hypermethylation on the cell lines exposed to inorganic arsenic. In contrast to the majority of animal and in vitro studies, human studies reported global hypo- and hypermethylation-induced arsenic exposure. Tellez-Plaza et al. [44] observed decreased global DNA methylation associated with a higher arsenic exposure level. Pilsner et al. [45] reported genomic methylation of PBL (peripheral blood leukocytes) DNA positively associated with plasma folate concentrations. They found that individuals with genomic hypomethylation of PBL DNA had a 1.8-fold increase risk for skin lesions, suggesting that changes in genomic methylation of PBL DNA may serve as an early biomarker of molecular events associated with the initiation and/or progression of As-induced skin lesions. Furthermore, these findings suggest that methylation of PBL DNA may serve as a functional biomarker to identify individuals at risk for future skin lesion development. In contrast, Majumdar et al. [46] indicated that arsenic levels in drinking water were associated with global DNA hypermethylation. In a case-control study, Pilsner et al. [47] showed an association between increasing global DNA methylation and urinary arsenic. Our findings confirmed that inorganic arsenic exposure in occupational settings (by inhalation) induces DNA hypermethylation.

Similarly to Niedzwiecki et al. [48], we did not find any significant correlation between age and 5-mC methylation, probably for the same reason, i.e., too small group in respect of age, in contrast to Pilsner et al. [45] observations. In our study, we found statistically significant differences between 5-mC % methylation in occupationally exposed group and controls, moreover smokers in occupational group showed a higher degree of 5-mC%. Generally, cigarette smoke impedes tracheobronchial clearance, possibly prolonging the exposure of bronchial epithelium to arsenic-containing dust particles, thus increasing the dose to the target pulmonary tissue [49]. Cigarette smoke is considered to be one of the most powerful modifiers of DNA methylation. In the present work, we observed increased hypermethylation in smokers, in contrast to another study where smoking is a proven factor limiting methylation, which is important in carcinogenesis [50]. A limited number of studies related to human exposure to arsenic and epigenetic modifications makes it difficult to explain this observation. Evidence from studies on populations exposed to As suggests a trend for an association between increasing arsenic exposure with increased DNA methylation although additional studies are needed to confirm those findings.

## Conclusions

The findings provide evidence showing inactivation of *NRF2-KEAP1* pathway in response to chronic arsenic exposure rather, but results should be interpreted with caution and necessarily confirmed by other studies in occupational exposure conditions. Moreover, occupational arsenic exposure is positively associated with global DNA methylation but the mechanisms are not clear yet. For this reason, further studies are necessary to determine the impact of exposure to arsenic on methylation and consequently the possibility to apply changes in DNA methylation as biomarkers of adverse health effects associated with iAs exposure.
